# Microbial Diseases of Bivalve Mollusks: Infections, Immunology and Antimicrobial Defense

**DOI:** 10.3390/md15060182

**Published:** 2017-06-17

**Authors:** Carla Zannella, Francesco Mosca, Francesca Mariani, Gianluigi Franci, Veronica Folliero, Marilena Galdiero, Pietro Giorgio Tiscar, Massimiliano Galdiero

**Affiliations:** 1 Department of Experimental Medicine—University of Campania “Luigi Vanvitelli”, Via Costantinopoli 16, 80138 Napoli, Italy; carlazannella88@gmail.com (C.Z.); gianluigi.franci@unina2.it (G.F.); veronica.folliero@unina2.it (V.F.); marilena.galdiero@unina2.it (M.G.); 2 Faculty of Veterinary Medicine, University of Teramo, Piano d’Accio, 64100 Teramo, Italy; fmosca@unite.it (F.M.); fmariani@unite.it (F.M.); pgtiscar@unite.it (P.G.T.)

**Keywords:** marine bivalve mollusks, antimicrobial peptides, bivalve immune system

## Abstract

A variety of bivalve mollusks (phylum Mollusca, class Bivalvia) constitute a prominent commodity in fisheries and aquacultures, but are also crucial in order to preserve our ecosystem’s complexity and function. Bivalve mollusks, such as clams, mussels, oysters and scallops, are relevant bred species, and their global farming maintains a high incremental annual growth rate, representing a considerable proportion of the overall fishery activities. Bivalve mollusks are filter feeders; therefore by filtering a great quantity of water, they may bioaccumulate in their tissues a high number of microorganisms that can be considered infectious for humans and higher vertebrates. Moreover, since some pathogens are also able to infect bivalve mollusks, they are a threat for the entire mollusk farming industry. In consideration of the leading role in aquaculture and the growing financial importance of bivalve farming, much interest has been recently devoted to investigate the pathogenesis of infectious diseases of these mollusks in order to be prepared for public health emergencies and to avoid dreadful income losses. Several bacterial and viral pathogens will be described herein. Despite the minor complexity of the organization of the immune system of bivalves, compared to mammalian immune systems, a precise description of the different mechanisms that induce its activation and functioning is still missing. In the present review, a substantial consideration will be devoted in outlining the immune responses of bivalves and their repertoire of immune cells. Finally, we will focus on the description of antimicrobial peptides that have been identified and characterized in bivalve mollusks. Their structural and antimicrobial features are also of great interest for the biotechnology sector as antimicrobial templates to combat the increasing antibiotic-resistance of different pathogenic bacteria that plague the human population all over the world.

## 1. Introduction

Marine bivalve mollusks may be affected by numerous infectious diseases. In this review, we will consider the most important diseases caused by viruses, bacteria and protistans, which are responsible for mortality outbreaks and have a substantial commercial impact. To a lesser extent, other diseases are also caused by fungi (*Aspergillus*, *Penicillium* and *Fusarium*) [1], Porifera (*Cliona* spp.) [2], and helminth parasites, such as trematodes, cestodes and nematodes [3].

Currently, the main infectious diseases of marine bivalve mollusks, such as herpes virus infection and bonamiasis, have taken on a worldwide distribution due to trade globalization. Since the transmission among bivalve mollusks is direct and horizontal, high-density production systems and environmental changes might have contributed to increasing the spread of diseases [4]; therefore, to avoid the current risk of further spreading of illnesses throughout the world, the World Organisation for Animal Health (OIE) *Aquatic Code* has set out standards and recommendations to improve the safety of international trade in aquatic animals, including marine bivalve mollusks. Nowadays, the aim is to prevent the pathogen’s introduction into an importing country, so as to avoid the onset of disease outbreak rather than to eradicate the pathogen, which would be more difficult and expensive because of the high-density production systems used in commercial hatcheries and nurseries and the continuous stock movements around the world. Due to the absence of effective and specific chemotherapy and anti-viral treatments or vaccines available to prevent illnesses [5], the surveillance and control plan of these diseases based on their prevention have a key role. For this reason, it is important to implement high levels of on-farm and live-holding facility biosecurity and to restrict stock movements. Avoiding stressors, such as exposition to intense temperatures, a high or low level of salinity, handling, substantial co-infection with other parasites, as well as decreasing density, should help to reduce the impact of diseases [4]. The present review will describe bacterial and viral pathogens that affect bivalve mollusks and will illustrate the immune responses generated in bivalves and the repertoire of immune cells and their activation upon infection. An aspect of importance in the immune defense mechanisms operated by bivalve mollusks is the expression of antimicrobial peptides; therefore, the second part of the review will be dedicated to providing an outline of the antimicrobial peptides that have been identified and characterized in bivalve mollusks.

## 2. Infectious Diseases of Marine Bivalve Mollusks

Taking into account all of these considerations, the development of reliable and useful diagnostic tools is of paramount importance for the prevention and control of diseases. Considering that the lack of bivalve molluscan cell lines has greatly limited the possibility for viral isolation and the study of the experimental transmission of these pathogenic microorganisms, currently, the detection of the causative agent of microbial disease is mainly based on direct diagnostic methods. Moreover, classic serological methods are not suitable for diagnostic purposes since mollusks do not produce antibodies; in fact, histology is considered as the standard screening diagnostic method because it supplies a wider amount of information, but macroscopic examination usually gives no pathognomonic signs or indicative information. It is difficult to diagnose an infection based exclusively on morphological differences between species that have similar morphological characteristics, such as for example *Bonamia ostreae* and *Bonamia exitiosa*, when observed under the microscope [6]. To overcome these problems, nowadays, more specific and sensitive molecular diagnostic techniques are used in addition to electron microscopy for specific identification of the pathogen. The OIE *Manual of Diagnostic Tests for Aquatic Animals* describes specific protocols designed to detect a certain pathogen agent, to be employed to confirm histological examination results and provides a species-specific diagnosis. Methods used in targeted surveillance programs should not be time consuming; in fact, in most cases, PCR and subsequently the sequencing of 16S or other candidate loci are recommended for the identification of the isolate. For a presumptive diagnosis of a disease, the standard method is histopathology, but for a confirmatory diagnosis, the standard methods are polymerase chain reaction (PCR) and in situ hybridization (ISH); however, sequencing and transmission electron microscope (TEM) are recommended.

Viral infectious diseases: the most important viruses associated with disease outbreaks and the major cause of mortality in bivalves are currently members of the families of *Herpesviridae* and *Iridoviridae*; nevertheless there are also other viruses that can infect bivalves belonging to the families of *Picornaviridae*, *Papovaviridae*, *Birnaviridae*, *Retroviridae* and *Reoviridae* [7]. Viral pathogens are often highly infectious and easily transmissible. High-density production systems and environmental changes might have contributed to increasing the spread of the disease [4]. Molecular tools such as PCR, ISH and immunochemistry are used to detect viral pathogens in mollusks [8,9,10].

Herpes-like viruses: The first description of herpes-like viral infection was reported by Farley et al. (1972) [11] in *Crassostrea virginica* from the east coast of the USA. Afterwards, disease outbreaks, associated with high mortality rates, particularly in larvae and spawn during the summer period, have been reported from Pacific oysters (*Crassostrea gigas*) in France, where higher mortalities were observed in 2008 and increased in 2009 and 2010 [12]. Oyster herpesvirus type 1 variant μvar (OsHV-1 μvar) was also found associated with Pacific oyster mass mortalities in Ireland, Italy, The Netherlands, Spain, the U.K. and in Australia, New Zealand and Korea, but is known to be detected elsewhere in the absence of oyster mortalities (e.g., Japan) [13]. Recently, OsHV-1 was detected in *C. gigas* in Japan and South Korea associated with mass mortality rate [14,15] and in Sweden and Norway [16]. In the Thau Lagoon (France), OsHV-1 and, secondarily *Vibrio splendidus* are responsible for mass mortality of *C. gigas* [17]. During the summer of 2012 and 2013, OsHV-1 caused high mortality of *Scapharca broughtonii* in China [10]. TEM analysis showed that larvae exhibited generalized infections, whereas focal infections usually occurred in juveniles. Adult stages were less sensitive than younger stages. Infected larvae showed a reduction in feeding and swimming activities, and mortality can reach 100% in a few days. The effects of the disease on the hosts manifested in velar and mantle lesions, and the attitude of larvae to swim weakly in circles. Histologically, fibroblastic-like cells exhibited abnormal cytoplasmic basophilia and enlarged nuclei with marginated chromatin; other cell types including hemocytes and myocytes showed extensive chromatin condensation [18].

Irido-like viruses: Maladie des branchies or gill disease is a disease caused by gill necrosis virus (GNV), responsible for recurrent mass mortalities in adult Portuguese oysters (*Crassostrea angulata*), from 1966 until the early 1970s along French coasts. To a lesser extent, gill disease also affected the Pacific oyster, *C. gigas*, imported in France, but with a negligible mortality. Because of its natural resistance to infection, *C. gigas* is currently the main species bred in Europe. Another Irido-like virus, hemocyte infection virus (HIV), caused mass mortality of Portuguese adult oysters (*C. angulata*) in France between 1970 and 1973. This virus is similar to GNV, and viral particles can be observed in the cytoplasm of atypical infected hemocytes in connective tissues. A third type of Irido-like virus, the oyster velar virus (OVV), caused a high mortality rate of Pacific larval oysters on the west coast of North America (Washington State, USA) from 1976–1984 [19]. Currently, Irido-like virus infections are uncommon in Europe.

Bacterial infectious diseases: Due to the marine bivalve filter-feeding habit, they concentrate a rich and diverse bacterial commensal microbiota, composed of various species belonging to different genera like *Vibrio*, *Pseudomonas*, *Acinetobacter*, *Photobacterium*, *Moraxella*, *Aeromonas*, *Micrococcus* and *Bacillus* [20]; some of them may be pathogenic in larval rearing systems and not in the wild; in fact, pathogenicity depends on the host species, their life stage, amount of bacteria and on environmental factors. The rate of larval mortality can reach 100%, especially if larvae are reared in static systems at high temperature and density. Diagnosis of bacterial diseases is based on macroscopic inspection of shell valves in combination with PCR, which is the most sensitive and rapid method [21].

Gram-negative bacteria: Most bacterial diseases of bivalves are caused by a large range of *Vibrio* species (*Vibrio alginolyticus*, *V. splendidus*, *Vibrio anguillarum*, *Vibrio tubiashi*, *Vibrio tapetis*, *Vibrio aestuarianuns*, *Vibrio neptunius* and other *Vibrio* spp.), *Pseudomonas* and *Aeromonas* [22,23,24,25]. These bacteria are responsible for bacillary necrosis in a wide range of species of bivalve larvae [26]. *Vibrio* spp. produce exotoxins (ciliostatic factors and hemolysins), which cause deciliation, loss of velar epithelial and abnormal swimming behavior [27]. Necrosis has been well described with histological, immunofluorescent and ultrastructure techniques [28]. *V. tapetis* is the activating agent of an epizootic infection described in adult clams called brown ring disease (BRD); the most sensitive species is *Ruditapes philippinarum* [21,29]. The disease has been detected in France, Spain, Portugal, Italy, the United Kingdom, Ireland, Norway [30] and, less frequently, in the Mediterranean and Adriatic seas [31]. In 2006, *V. tapetis* was reported from Manila clams on the west coast of Korea [32]. Transmission passes through direct contact between infected clams [33]. *V. tapetis* adheres to and colonizes the surface of the periostracal lamina at the mantle edge of the shell, causing anomalous deposition of periostracum, an abnormal calcification process and the accumulation of brown organic material, which is a striking sign of the disease. From the extrapallial space, the bacteria can penetrate the mantle epithelium and the soft tissues, where they reproduces themselves and cause severe damage and subsequent death [34]. Juvenile oyster disease (JOD) is a similar syndrome to BRD that appeared in 1988 in juvenile *C. virginica* [35] caused by *Roseovarius crassostreae*; nowadays, the disease is known as *Roseovarius* oyster disease [36]. Major mortal outbreaks have been reported in cultured oysters from New York to Maine (USA). Symptoms, such as reduced growth rates, fragile shell development, cupping on the left valve, anomalous conchiolin deposit around the periphery of the mantle on the inner valves [37], occur when water temperature exceeds about 21–25 °C, in high salinity and high-density culture conditions. Mortality reaches up to 90% in animals <25 mm; instead, larger juveniles forms show lower rates of mortality [38].

Gram-positive bacteria: Few Gram-positive bacteria cause diseases in bivalves; the main pathogenic agent is represented by *Nocardia crassostreae*, the etiological agent of Pacific oyster nocardiosis (PON) infecting *C. gigas* and *Ostrea edulis* cultivated near infected *C. gigas* along the west coast of North America from the Strait of Georgia, British Columbia to California and Japan (Matsushima Bay) [39]. Carella et al. (2013) [40] have notified nocardiosis in Mediterranean bivalves. Mortality rate reaches up to 35%; infected animals show yellow-green pustules in the mantle, gills, adductor and cardiac muscle associated with intense hemocyte infiltration around the colonies of *Nocardia* [41].

Protozoan infectious diseases: The most important protozoan pathogens belong to the genera *Bonamia*, *Perkinsus*, *Haplosporidium* and *Marteilia*; they can mainly infect oyster and clam species, causing enormous damage to commercial productions. In particular, in this review, we will discuss in detail the diseases caused by *Perkinsus marinus*, *Perkinsus olseni*, *Marteilia refringens*, *B. ostreae* and *B. exitiosa* that are currently under surveillance and require mandatory notification by the World Organization for Animal Health. Prevalence and intensity of infections tend to increase during the warm season, depending on temperature and high salinity rates [42]. For correct identification of the pathogen, histological examination is needed, as well as ISH or PCR. However, for unknown susceptible species and unknown geographical range, confirmation by sequencing and description by TEM are recommended. Bonamiosis represents 63% of protozoan diseases in Europe [43] and is caused by a group of protists in the genus Bonamia. *B. ostreae* has spread in Europe (France, Ireland, Netherlands, Portugal, Spain and the U.K.), but also on the west coast of Canada and both coasts of the USA [44]. *B. ostreae* was found in *O. edulis* imported to China [45]; recently, this species was detected in New Zealand infecting the flat oyster *Ostrea chilensis* [46]. *B. exitiosa* was found in *O. chilensis* in South Island, New Zealand [47]*,* and in *Ostrea angasi* in southeastern Australia [48]. Since 2003, the parasite has been observed in both the Atlantic and Pacific coasts of the USA [49], including California [50]; *B. exitiosa* was also detected in *O. edulis* from the Galician coast (Spain) and the Manfredonia Gulf, Italy (Adriatic Sea), including concurrent infections with *B. ostreae* [51] and in *Ostrea stentina* in Tunisia [52]. *B. exitiosa* was found, as well, on the Spanish Mediterranean coast [53], in southwestern England [54] and in southern Portugal [55]. Others species, *Bonamia perspora* and *Bonamia roughleyi*, have been found on the east coast of the USA [56] and in southeastern Australia [57]. With the exception of *B. perspora*, all of the other species can be normally observed within hemocytes of the host [58]; *B. perspora* occurs within connective tissues [56]. The pathogen infects the hemocytes, multiplies within blood cells and spreads to all tissues. In highly infected adult oysters, we can find a yellow discoloration of the tissue, extensive lesions on the gill and mantle, breakdown of connective tissue and significant mortality (>90%). Larvae can be infected and contribute to the spread of the parasite. A lack of resistance to infection and, therefore, high densities of oysters in closely-spaced beds favor the development of epizootics.

In the late 1960s, two protozoans, both in the genus *Marteilia*, *Marteilia refringens* and *Marteilia sydneyi*, were identified as the causative agents of disease and heavy mortalities in the flat oyster: *O. edulis*, in France, and in *Saccostrea glomerata*, in Australia, respectively [59]; afterward, the parasite become widespread: *M. refringens*, infecting *O. edulis*, has, to date, mainly been found in Europe (Albania, Croatia, France, Greece, Italy, Morocco, Portugal, Spain, Sweden, Tunisia and the United Kingdom); *Mytilus galloprovincialis*, in the Gulf of Thermaikos, northern Greece [60], on the north coast of the Adriatic Sea [61] and along the Campanian coast (Tyrrhenian Sea, South of Italy) [62]. Marteilia was also previously found in *M. galloprovincialis* bred in Puglia [63]. *M. sydneyi*, infecting *Saccostrea glomerata* and possibly other *Saccostrea* spp. [64], has been reported in New South Wales, Queensland and Western Australia [65]. Marteiliosis is also known as Aber disease (*M. refringens*, two types: M and O, as defined by Peruzzi et al. [66], and Queensland Unknown (QX) disease (*M. sydneyi*). Both infect the digestive system and sporulate in epithelial cells of the digestive gland causing paleness of the digestive glands, emaciation of the oyster, dissipation of its reserves of energy, tissue necrosis, cessation of growth and mortality up to 90% in summer. Juveniles and older life stages are susceptible to infection, but prevalence and infection intensity are generally higher in individuals of two years old or more [67]. By TEM analysis, *M. sydneyi* can be differentiated from *M. refringens* by a paucity of striated inclusions within the plasmodia, the formation of eight to sixteen sporangial primordia in each plasmodium (instead of eight for *M. refringens*), the occurrence of two or three spores in each sporangium (rather than four in *M. refringens*) and the presence of a thick coat of concentric biological membranes surrounding mature *M. sydneyi* spores.

Perkinsosis: Perkinsosis is caused by *Perkinsus marinus*, responsible for dermo disease in *C. virginica* and, to a lesser extent, in *C. gigas*, *Crassostrea rhizophorae* and *Crassostrea corteziensis* [68]. The pathogen, uncommon in Europe, was first described in the Gulf of Mexico [69]. It was found along the southeast coast of the USA from Maine to Florida [70], along the Pacific coast of Mexico [71], in the Gulf of California (northwest Mexico) [72] and in Brazil [73]. *Perkinsus olseni* causes perkinsosis in many clam species with distribution in Australia, Korea, China, Japan and Europe [32]. Both parasites were also found associated with ten oyster species collected from both Panamanian coasts, including the Panama Canal and Bocas del Toro [74]. Transmission is direct from oyster to oyster; viable cells are released in host feces or on the death of the host [32] and are acquired through host feeding mechanisms. Every life stage is susceptible to disease [75]. The pathogen infects and proliferates in the digestive epithelium, connective tissue of all organs and hemocytes causing hemocytosis and tissue lysis with a consequent severe emaciation; mortality arrives up to 80% based on environmental factors [76]. In order to simplify all microbiological diseases, we generated tables with details of the infections (Table 1, Table 2 and Table 3).

In conclusion, we must as well remember that for a disease to occur, the synergy among three factors is required. This synergy is commonly named the epidemiological triangle, which is composed by a host, a pathogenic agent and the environment. In fact, some of the infectious agents may be pathogenic or nonpathogenic based on the host species, its life stage (larval, juvenile or adult form) and the immune system, on which the environmental factor plays a key role. We can, therefore, assert that, in general, the exposition to extreme temperatures, a too high or too low level of salinity, human handling, an increasing density in rearing systems and co-infection with other parasites may reduce the immune defenses of the host, as well as increase the pathogenic agent rate of growth and, hence, its pathogenicity, making the host more susceptible to illness.

Based on the knowledge we have today on how a disease can spread and acknowledging the key role of the environment, it is clear that an improvement of the surveillance on the environment of rearing systems is essential.

## 3. Defense Mechanisms in Marine Bivalve Mollusks

During the last few decades, the immunology of marine bivalve mollusks (MBM) has been investigated with great interest, leading to the development of different branches for basic and applied research. The hemocyte phagocytosis constitutes the major immune response in MBM, and the study of this highly conserved process has contributed to better understanding not only the pathogenetic mechanisms of infectious diseases in MBM [81], but also the role of the filter-feeding organisms as passive carriers of pathogens to humans, considering the involvement of the hemocytes and hemolymph factors on the microbial clearance from mollusk’s tissues [82].

The study of the hemocyte properties as biomarkers for monitoring the biological effects of anthropogenic stressors in polluted sites [83], as well as measuring the economic impact of environmental stressors on shellfish productions [84] represents another interesting immunological field of investigation for MBM. Moreover, the basic investigation of the hemocyte/hemolymph system represents a simplified phylogenetic model for understanding the ancestral interactions and integrations that occur between immunity and neuroendocrine response [85] and, more generally, between defense mechanisms and host homeostasis.

In the present section, we have mainly described the most important phases that characterize the hemocyte phagocytosis in MBM, giving, in parallel, importance to the humoral factors that participate in the recognition and opsonization of foreign particles, thus focusing on the common features of the innate immunity that are shared by invertebrate hemocytes and phagocytic cells of higher vertebrates.

**Figure 1:**
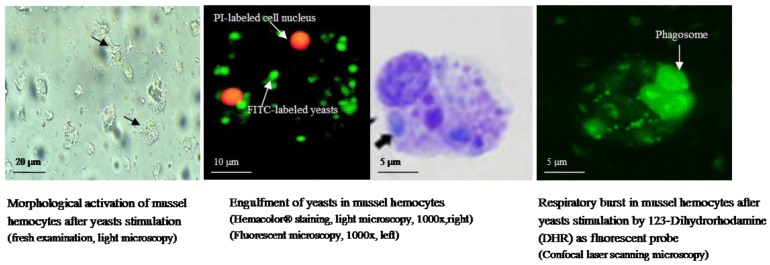
The figure reports the main phases of the hemocyte phagocytosis, as well as previously investigated in mussel (author’s unpublished figures) that are particularly described in the present section, in combination with the main humoral opsonizing and degradative factors.

Hemocyte-mediated immunity: The cell-mediated immunity represents the main internal defense response of marine bivalve mollusks. The hemocyte phagocytosis constitutes the key activity, leading to the recognition, engulfment and demolition of biotic and abiotic foreign particles [86]. The innate immune properties of the hemocytes rely on their ancestral role in food digestion and nutrient transport [87]. Indeed, the interplay between phagocytosis and nutrition in invertebrates has been ascribed to the primary function of the hemocytes phagosome as the digestive organelle, where microorganisms are degraded as nutrients source, then evolving into a more specialized compartment to kill pathogens (Figure 1) [88].

Marine bivalve mollusks possess an open circulatory system, and the hemocytes are found either in hemolymph or in tissues, respectively as circulating or infiltrating cells [89]. From an ontogenetic point of view, some authors suggested that hemocytes are derived from connective tissue cells [90]; however, different models have been proposed about the types of progenitor cell lines [91]. The nomenclature of mollusks hemocytes still represents a subject of debate, and the efforts at developing a uniform classification have resulted in the recognition of two main types of cells, such as granulocytes and hyalinocytes, based on morphological appearance and granularity under microscopic examinations. However, flow cytometry [92], electron microscopy [93,94] and monoclonal-antibodies based assays [95] have suggested the presence of various hemocyte sub-types. Such diversity may reveal a broad array of activities, and in particular, it is widely accepted that granulocytes play the most active role in the phagocytosis response [96]. Nevertheless, all of the hemocyte populations contribute to the overall immune response, operating in a differential fashion on the basis of the different stimulations [97]. The relative concentration of the various circulating hemocyte types can be exposed to reversible and selective modifications through physiological and molecular mechanisms comparable to the margination/demargination processes, which take place in humans and other mammals [98]. Therefore, the quantitative diversity of the cellular hemolymph configuration represents an important factor for the modulation of the immune response [99].

The interest for the study of the hemocyte immunity mainly derives from the role of marine bivalve mollusks as sentinel organisms in environmental monitoring [100]. Indeed, many hemocyte parameters have been investigated as biomarkers in field or laboratory studies [101,102,103], and detailed data are available in the literature about the strong influence of natural and anthropic stressors on the hemocyte activity. In particular, the disruption of their morpho-functional properties has been described after exposure to low [104] and high [105] temperatures, pH acidification [106], mechanical stress [107], salinity changes [108], exposure to air [109], harmful algal bloom [110], organic and inorganic contaminants [111]. Nevertheless, some endogenous factors seem to have also an important influence on the modulation of the hemocyte activity, such as age [112], gender [113] and reproductive stage [114].

The hemocyte phagocytosis, mechanisms and kinetics: Bivalve hemocytes resemble the vertebrate monocyte/macrophage lineage, both in structure and function [115]. The chemotactic ability of hemocytes to migrate toward foreign particles and to incorporate them inside phagosomes is closely dependent on morphological activation through the projections of membrane ruffles or pseudopodia [116]. The cytoskeleton re-arrangement following proper stimulation represents the pivotal mechanism that allows the hemocytes to acquire a morphological spreading, from roundish to irregular shape [117]. Previous studies demonstrated that bacterial products, such as lipopolysaccharides and formylated tripeptide (N-FMLP), were able to elicit chemotactic and/or chemokinetic reactions in hemocytes [118], and the type of cell movement appeared as dependent on the nature of chemoattractant, thus hypothesizing a receptor-dependent mechanism [119]. Indeed, a differential migration activity was detected in hemocytes on the basis of the bacteria types that were encountered [120]. Both chemotaxis and chemokinesis augment the probability of physical association between hemocytes and foreign particles, but to date, the knowledge about pattern recognition receptors (PRRs) is still limited in bivalve hemocytes. PRRs recognize the conserved highly repeated microbial structures, termed pathogen-associated molecular patterns (PAMPs), and the Toll-like receptors (TRLs) have a prominent role within PRRs group, being traced to the most ancestral multicellular invertebrates [121]. TLRs belong to type I membrane receptors and contain an extracellular leucine-rich repeat (LRR) domain mediating the recognition of PAMPs [122]. Unlike mammal TLRs, invertebrate TLRs could not directly recognize PAMPs, but they seem to require the cytokine-like molecule Spatzle as an assistant [123]. Indeed, some authors suggested a hybrid function in pattern recognition for the primitive mollusk TLR, being characterized by broader ligands affinity and involving the assistance of some serum components [124].

In the Pacific oyster *C. gigas*, a putative TLR was cloned and named CgToll-1, showing upregulation in the hemolymph after challenge with *V. anguillarum* [125]. Similarly, in the scallop *Chlamys farreri*, a Toll homologue was detected and named CfToll-1, revealing transcripts modulation in the hemocytes after exposure to LPS [126]. In the mussel *Mytilus edulis*, the transcriptome analysis indicated a wide repertoire of innate recognition receptors, including transcripts for 27 TLR, particularly expressed in hemocytes [127]. Following binding of the ligand to the extracellular segment of TLR, signal transduction takes place by the intracellular toll-interleukin domain (TIR) containing adaptor molecules [128]. Each TLR recognizes distinct microbial components and activates different signaling pathways using selected adaptor molecules, then leading to the engagement of the signaling cascade of protein kinases that ultimately activate transcription factors and the expression of genes involved in the immune response [129,130]. In contrast to the large amount of data on TLR signaling systems from higher vertebrates, relatively little is known in bivalve mollusks. The existence of genes/transcripts mediating the Toll signaling pathway in hemocytes was reported in *M. galloprovincialis*, showing upregulation after bacterial challenge, particularly by Gram-negative, whereas a marginal response was detected following stimulation with purified PAMPs (LPS, β-glucans) [131]. Intermediate transcripts of the Toll signaling pathway were also detected in scallop [132], clams [133] and oysters [134]. Moreover, the intensity and duration of intermediate components activation, such as kinase-mediated cascade, appeared as dependent on the type of extracellular stimuli [135]. This kind of evidence contributes to support the existence of a differential hemocyte response depending on the bacteria types that are used for challenge [136]. Although most of the studies focused on the presence of Toll pathways in marine bivalve mollusks, other types of receptors have been investigated. Recently, the mRNA transcripts of a new putative phagocytic receptor (C*g*Nimc), belonging to the Nimrod superfamily, were identified in hemocytes of *C. gigas*, revealing upregulation after bacterial challenge, whereas the recombinant protein showed higher binding affinity toward LPS rather than peptidoglycan [137]. In the scallop *Argopecten irradians*, a peptidoglycan recognition protein (PGRP) was cloned, sharing high identity with PGRPs of higher organisms and showing upregulation in the hemocytes exposed to peptidoglycan, but not to LPS [138]. Two short PGRPs were also detected in the bivalve *Solen grandis*, and they were particularly induced by peptidoglycan and β-1,3-glucan [139]. Following an engulfment of foreign particles within hemocyte phagosome, the activation of lysosomes granules leads to the formation of phago-lysosomes vacuoles where intracellular digestion takes place [140]. The lysosomal enzymes strongly participate in the degradation of ingested, material and the hydrolytic activity of β-glucuronidase, phosphatases, esterases and sulfatases has been detected in mussels [141], clams [142], cockles [143] and oysters [58]. Oxidative enzymes, such as peroxidase and phenoloxidase, are also involved in degradative mechanisms, but their presence is not a common feature in any marine bivalve mollusks [144,145]. The respiratory burst represents another heavy microbicidal mechanism, and the generation of the highly oxidant reactive oxygen species (ROS) inside phago-lysosomal vacuoles of stimulated hemocytes was suggested as an NADPH-oxidase-dependent mechanism [146,147,148]. The ROS synthesis has been widely reported in mussels [149], oysters [150], scallops and clams [151], although some authors indicated the lack of the NADPH-oxidase activity in the family Veneridae. The detection of ROS has been mostly investigated in terms of defense mechanism; however, the role of these molecules has been also considered in cellular and tissue homeostasis [152]. Indeed, previous reports indicated the ability of mussel hemocytes to generate ROS in the absence of phagocytic stimulation [153], and more recent evidence has suggested that mitochondria represent the main source of ROS in the unstimulated hemocytes, rather than the activity of lysosomal NADPH-oxidase [154].

Humoral defense factors: The hemocyte degranulation and the extracellular release of lysosomal enzymes represent the first humoral defense mechanism that was investigated in marine bivalve mollusks [155], a strategy commonly described as a response to pathogens [156]. However, marine bivalve mollusks possess more selective extracellular tools to contrast invaders, including recognition and effector proteins, such as lectins, complement-like molecules, lipopolysaccharide- (LBP) and β-1,3-glucan-binding proteins (β-GBP), fibrinogen-related proteins (FREPs) and antimicrobial peptides (AMPs) [156,157,158]. Lectins represent carbohydrate-recognition proteins, and their agglutinating and opsonizing activities have been previously described in marine bivalve mollusks, revealing heterogeneous binding specificity towards microbial surface sugars [159,160]. In particular, C-type lectins can recognize and bind terminal sugars on glycoproteins and glycolipids in a calcium-dependent manner. Recent studies in different marine bivalve mollusks have demonstrated both their gene upregulation following bacterial challenge and the binding activity of the recombinant proteins towards purified PAMPs [161,162,163]. Galectins, formerly known as S-type lectins, represent another conserved and ubiquitous family of carbohydrates-binding proteins, particularly characterized by their affinity for β-galactosides [164,165,166,167]. In the clam *R. philippinarum* [168] and scallop *A. irradians* [169], galectins have been cloned and characterized, showing gene upregulation and agglutination activity following bacterial challenge. Galectins seem to possess also an opsonizing role by promoting the hemocyte phagocytosis through cross-linking between extracellular glycocalyx and hemocyte surface, as observed for the oyster galectin CvGal1 [170]. Homologues of the vertebrate complement cascade have been investigated in marine bivalve mollusks for their immune role against pathogens. C1q represents the first sub-component of the classical complement pathway, and to date, C1q domain-containing proteins have been characterized at molecular level in oysters [171], mussels [172], scallops [173] and clams [174], revealing high molecular diversification of this family [175]. From a functional point of view, the recombinant proteins showed binding activity towards whole bacterial cells, as well as isolated PAMPs [176]. In addition, some authors have identified in oyster a complement component C3-like gene, particularly expressed in the hemocytes [177]. Although AMPs represent the most examined group of antimicrobial proteins, further discussed in the present review, other bactericidal compounds have been identified in marine bivalve molluscan integrative components of their humoral defense system. Member homologues of the bactericidal/permeability-increasing protein (BPI) family were isolated, showing binding activity toward LPS and bactericidal properties against Gram-negative bacteria [178,179,180]. Members of the lysozyme families (N-acetylmuramide glycanhydrolase) have been characterized in mucosal tissues and secretions of several bivalve species, as described in mussels [181], clams [182], scallops [183] and oysters [184], displaying a broad spectrum of antimicrobial activity and playing a dual role both in nutrition and immunity [185]. Moreover, the presence of plasma proteases’ activity was previously described in marine bivalve mollusks as a microbicidal mechanism [186,187], and such evidence has accounted for the isolation and characterization of genes encoding proteases, such as cathepsins [188].

In conclusion, the great part of the studies on bivalve immunity has been directed to investigating the morpho-functional properties of circulating hemocytes and the humoral defense factors, providing limited information about the spatial and temporal heterogeneity of the immune response. In the future, a better understanding of microbe-bivalve interactions at mucosal interfaces is required, considering the interplay between mutualistic, commensal and pathogenic microbes at the initial encounter/colonization sites [189].

## 4. AMPs and Their Mechanism of Action

Marine ecosystems constitute more than 70% of the Earth’s surface, are associated with astonishing species diversity and, therefore, represent an enormous resource of pharmacologically-active molecules. Marine living beings can be reconsidered as a potentially unlimited reservoir of bioactive molecules, either derived by complex metabolic reactions or gene-encoded peptides [190,191]. For each milliliter of seawater, approximately 10^6^ bacteria and 10^9^ viruses are generally present; therefore, seawater is to be considered an abundant source of pathogens. Most marine organisms reside in intimate coexistence with pathogenic microbes, and their survival in such a hostile surrounding is directly dependent on the development of a vigorous and successful immune system. In fact, living marine organisms are continuously exposed to microbial hazards, and to maintain their safeguard in such a harsh environment, they need a strong defensive mechanism to control all microbial pathogens that are inglobated with nutrients [192,193]. In fact, microbes are accumulated in bivalves, and microbial densities in their tissues are generally greater than in seawater. If the filtered microbes are pathogenic, their concentration in bivalve tissues can be deleterious. In order to defend themselves against such detrimental pathogens, bivalves depend on cellular defense mechanisms, as earlier described, and humoral defense factors, among which AMPs play an important role.

As a matter of fact, bivalves having evolved in the constant proximity of microorganisms must rely on their innate immune system effector molecules to contrast microbial pathogens. An ancient mechanism of innate immunity is represented by the production of anti-microbial substances, primarily peptides or polypeptides, which are produced by different types of cells and secretions and are either constitutively synthesized or induced at the time of infection [191]. In this regard, marine bivalves represent a valuable and scarcely delved source for novel antimicrobials [194].

Since the innate immunity system is supposed to represent the primary line of host defense against invading pathogens, it is of paramount importance to maintain host-microbe homeostasis and AMPs as ancient evolutionary molecules universally distributed in most of the multicellular organisms, which perform a broad-spectrum antimicrobial activity and, often, also present an immunomodulatory capacity. Therefore, AMPs play a crucial role in host defense against a wide range of microorganisms including Gram-positive and Gram-negative bacteria, viruses, fungi and parasites. Since their first discovery, with the isolation of a peptide named cecropin [195,196,197], from the insect *Hyalophora cecropia*, almost 2000 sequences encoding putative AMPs have been described and included in “The Antimicrobial Peptide Database” (http://aps.unmc.edu/AP/main.php).

**Figure 2:**
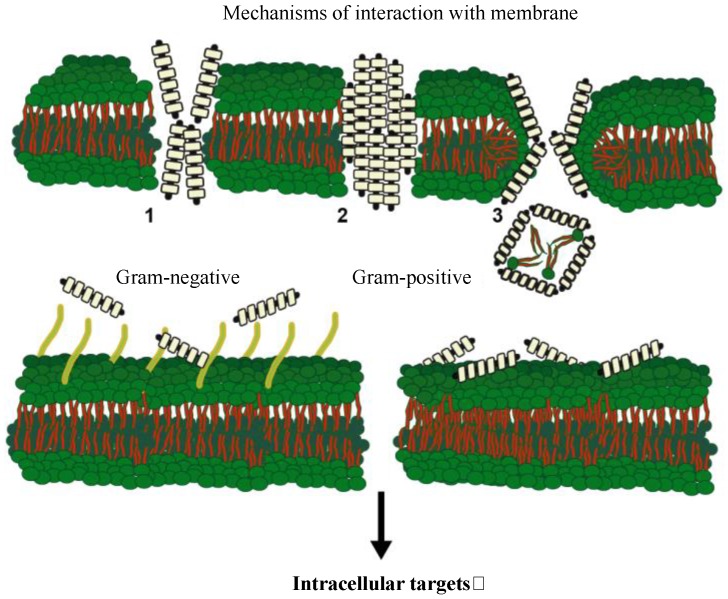
Mechanisms of interaction of AMPs with membranes. Top: The main proposed modes of action are: carpet model (1), barrel stave model (2) and toroidal-pore model (3). Bottom: Interactions of AMPs with Gram-negative and Gram-positive bacteria.

AMPs are relatively small peptides (<60 amino acids) and may play polyvalent roles, which expand beyond their ability to serve as antibiotics [198]. Several of these peptides have been proven to possess anticancer activity, to be able to stimulate the immune system by promoting cytokine release, promote chemotaxis, antigen presentation, angiogenesis, inflammatory responses and adaptive immune induction [199,200,201]. During the last few decades, they have been purified from plants, invertebrates and vertebrates and are consequently considered to be part of the immune process probably in all Metazoa, representing innate immunity actors conserved along evolution in all biological kingdoms [202,203,204]. Despite their ample variation in biophysical characteristics, such as mass, composition and primary structure, several functional correlates have been identified [205]. In fact, the embracing features of most AMPs include rapid killing mechanisms, broad spectra of action, a clear net cationic charge and a strong propensity to give rise to amphipathic surfaces able to promote peptide: membrane interactions [206]. Although the primary structures of AMPs are diverse, based on genomic and protein sequences analysis coupled with structural and functional studies, AMPs have been sorted into several groups, including: (i) linear peptides able to adopt an α-helical conformation in a membrane-mimetic environment, (ii) peptides stabilized by one or several pairs of cysteine residues able to form disulfide bridges that have structures predominantly composed of β-sheets, (iii) peptides with a high content of specific amino acids, such as prolines, arginines, tryptophans, histidines and glycines, but with no uniform secondary structures, and (iv) peptides derived by partial hydrolysis of bulky precursor proteins with unknown or limited antimicrobial activities before the enzymatic degradation. AMPs’ antimicrobial activity derives from membrane disruption and osmotic lysis of bacteria as opposed to the usual mechanism of action of most antibiotics where specific sites during bacterial growth and replication are targeted. Moreover, some AMPs proved to be also efficient in inhibiting viral infections. The putative mechanism for exerting an antiviral activity seems to be: (i) blocking early steps of viral entry by surface carbohydrate interaction, (ii) blocking viral attachment or penetration into the host cells by interactions with specific cellular receptors, (iii) interaction and inactivation of viral envelope glycoproteins, (iv) modulation of host cell antiviral responses, (v) blocking intracellular expression of viral genes and/or production of viral proteins. However, no unequivocal correlation between AMP structures and microbial inhibition or killing mechanisms has so far become obvious; in fact, striking differences from peptide to peptide and specificity for particular AMP-microbe combinations are generally observed. The forthright antibiotic action of AMPs is considered to hinge on their cationic and amphiphilic nature, which empowers these molecules with the ability of interact with negatively-charged bacterial surfaces and membranes, therefore leading to membrane disruption or alteration [207]. In fact, AMPs essentially take advantage of the broad differences found in the organization of bacterial against eukaryotic membranes in order to promote damage of the membrane. Several differences, such as the absence of cholesterol, the abundance of anionic lipids and an electric field with a strong inward direction, are decisive for the correlation of specificity in favor of AMPs action against bacterial pathogens and a lower toxicity toward host cells [208,209]. Moreover, it is unlikely that bacteria can spoil these features and develop resistance since it would necessitate a profound modification of the bacterial membranes and their functions. Even though the exact AMP mechanisms of action remain a subject of discussion, the majority of them share similar biophysical characteristics that allow peptides to interact with microbes. Importantly, AMPs’ antibacterial power is directly attributable to some of these features, such as a net positive charge, enabling AMP-bacterial membrane interactions via electrostatic forces and the propensity to form amphipathic structures in hydrophobic environments allowing penetration into the bacterial phospholipid bilayer. Therefore, regardless of any variations in size and structure, AMPs are often portrayed with an intrinsic cationic and hydrophobic nature that is the key for their first interaction with target bacterial cells. In agreement with literature data, AMPs’ mode of action seems to proceed similarly to a pore-forming action or to a detergent effect. Several models have been put forth to explain such mechanisms, namely the barrel-stave model, the toroidal model and the carpet-like model (Figure 2) [210]. The three models were all elaborated following the assumption that AMPs have the tendency of being attracted by the bacterial membrane in virtue of electrostatic bonding forming between the peptide cationic feature and the low electric charge conferred to outer bacterial membranes by their surface components, such as phosphate groups within the lipopolysaccharide (LPS) of Gram-negative bacteria or lipoteichoic acids abundant on the exterior of Gram-positive bacteria [211].

These models can be described in brief as follows: in the barrel-stave model, peptides (more often α-helical peptides with marked hydrophobic-hydrophilic domains), once attached to the phospholipids, aggregate and enter inside the membrane bilayer with the hydrophilic peptide domains forming the inside of the pore, and the hydrophobic peptide parts lined up facing the lipidic region of membrane phospholipids [212]. As a result, transmembrane pores, made up of a bundle of amphipathic helices, are created in a perpendicular topology within the membrane. A toroidal model with lipids intercalating between helices can be envisioned when peptides remain linked with lipid head groups, also when peptides are located upright within the highly-curved lipid bilayer. In this case, diffusion of lipids between the outer and inner membrane layers is granted by the continuous surface formed by both membrane leaflets [203,213]. A carpet-like model revealed instrumental for describing AMPs mechanism devoid of the straight insertion within the hydrophobic core of the membrane, but with peptides accumulation in an oriented array on the membrane surface forming a real carpet-like structure. When a threshold concentration is attained, the formation of transitory pores with disruption of lipid assembly and a detergent-like cell lysis ensues. This also leads to the micellization of the bilayer. Hence, all models considered, membrane perturbation is driven by cationic and hydrophobic residues shaping the interactions between peptides and phospholipids [214]. Nevertheless, an oversimplification of the hypothetical pore models is generally applied to the description of the mechanisms of action of AMPs [215]. More likely, a disordered or chaotic pore can be envisaged where peptides may shift their conformation, mutating the charges and relative interactions with lipids and, therefore, allowing the flickering of pores [216].

In conclusion, the interfacial activity is a leading determinant of the permeabilizing activity of several peptides [217], and it also provides a useful means to differentiate between peptides with antibacterial power that have a detrimental effect on membrane bilayers and cell-penetrating peptides, which seem to move past the bilayer without producing serious damages [218,219]. A further possibility for the antibacterial mechanisms is the action AMPs can exert on microbial intracellular targets where peptides can block cell-wall and/or nucleic acid synthesis, protein production and enzymatic activities [203].

## 5. Marine Bivalve Antimicrobial Peptides

Marine AMPs have been discussed thoroughly in other reviews [220,221,222,223]. However, in the present review, we describe AMPs derived from marine mollusks and their application in fighting infectious diseases. One unanswered enigma remains: the understanding of how mollusk survive in the absence of an acquired immune system since they are in close contact with a magnitude of putative pathogens such as viruses, bacteria, fungi and parasites, as a consequence of their filtering activities. Fortunately, mollusks encode for several antimicrobial molecules, and several AMPs have been isolated from marine mollusks, such as mussels (major species analyzed: *M. galloprovincialis*, *M. edulis*), clams (major species analyzed: *Venerupis philippinarum*), scallops (major species analyzed: *A. irradians*, *Argopecten purpuratus* and *Chlamys nobilis*) and oysters (major species analyzed: *C. virginica* and *C. gigas*).

**Figure 3:**
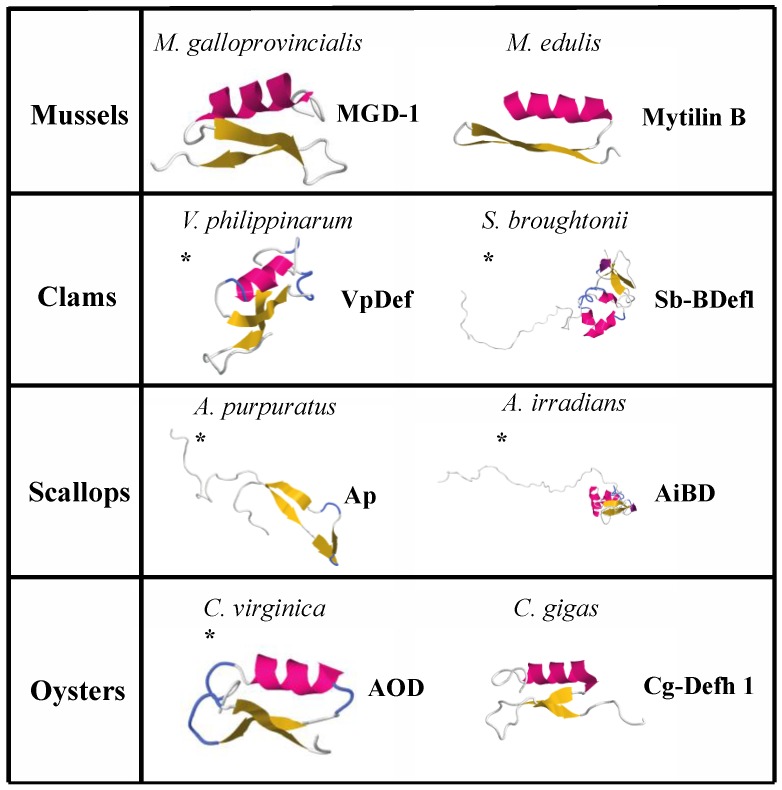
Examples of three-dimensional structures of bivalve AMPs. * The predicted structures were designed using AIDA (ab initio domain assembly) server, a tool for the prediction of protein tridimensional structures (http://ffas.burnham.org/AIDA).

Initial identification of AMPs in bivalves dates almost 20 years ago with the pioneering studies that led to the characterization of the first AMPs in mussels [224,225,226,227]. To date, several AMPs have been described from mussels and other bivalves. The majority of them rank along to the group of cysteine-containing peptides, which include a huge variety of defensins and defensin-like peptide and larger proteins. The discovery of the first molluscan AMP was performed by Hubert et al., 1996, from the *M. galloprovincialis*. Subsequently, several AMPs have been identified and extensively studied in two main mussels species, *M. galloprovincialis* and *M. edulis*. These molecules with antimicrobial properties have been classified into four groups following a primary structure classification parameter: defensins, mytilins, myticins and mytimycin. Overall, mussels’ AMPs are characterized by possessing strong hydrophobic and cationic properties and a signature amphipathic structure (α-helix, β-hairpin-like β-sheet, β-sheet or α-helix/β-sheet mixed structures), all considered fundamental for the antimicrobial activity displayed. The principal defensin from mussels is a 39-long peptide present in two isoforms MGD1 (PDB 1FJN) and MGD2 sharing significant sequence homology. Furthermore, the three-dimensional structure (Figure 3 shows the 3D structure of several mollusk-derived AMPs) has been solved using NMR analysis [228] and has shown the presence of a side helical part (spanning from Asn7 to Ser16) and two anti-parallel β-strands (spanning fromArg20 to Cys25 and from Cys33-Arg37), which constitute the common cysteine-stabilized motif.

A similar structural icon has been recently described in the attempt to unify all known classes of Cys-stabilized antimicrobial peptides. Yount and Yeaman identified this common structural signature and named the “γ-core motif” [229,230]. Conservation of the γ-core motif across all living organisms suggests it may represent an antimicrobial peptide archetype; in fact, several structural topologies resembling a γ-core motif can be described in a wide range of organisms, from unicellular organisms to humans. The γ-core present in many AMPs is not the only structural determinant to confer an antimicrobial activity, but in many instances, it can be used as a scaffold, to which further antimicrobial determinants (e.g., α-helices or β-sheets) can be attached in a modular fashion to yield various configurations. Higher organisms showed the most diversified range of γ-core polypeptides. This is, though, expected considering the necessity to provide protection to diverse tissues and to cooperate with other useful immune-system components. For example, several studies have been recently conducted in the analysis of such determinant in human β-defensins (HBDs), and the γ-core alone has been proven sufficient for retaining substantial antimicrobial activity [231,232,233,234]. Nevertheless, there is a significant difference with most of the know AMPs bearing the γ-core signature; that is, the fact that mussels defensins are characterized from being stabilized by four disulfide bonds (Cys4-Cys25, Cys10-Cys33, Cys14-Cys35 and Cys21-Cys38 in MGD-1), instead of the three disulfide bonds generally described in most other molecules, including arthropod defensins. MGD1 [224], a member of the arthropod defensin family from edible Mediterranean mussels (*M. galloprovincialis*), and MGD2 [227] share the same size and sequence showing 80% identity with amino acids. Both contain an ORF encoding 81 amino acids including a 21-residue N-terminal sequence with a highly hydrophobic core representing a signal domain, followed by a 39-amino acid sequence corresponding to the active defensin and a 21-residue C-terminal extension. MGDs are principally active against Gram-positive bacteria, but MGD2 showed increased activity also against Gram-negative bacteria. Structural features of MGD1, cardinal for the supply of antimicrobial activity, were analyzed by Romestand et al., 2003 [235], by producing a set of synthetic peptides analogous to the described secondary structures of the molecule. The nonapeptide from residue 25 to residue 33 (CGGWHRLRC) displayed a consistent bacteriostatic activity, especially when cyclized by a disulfide bridge between Cys25 and Cys33.

The second group of mussels-derived AMPs is represented by the mytilins family, comprising five isoforms (A, B, C, D and G1). Isoforms A and B were found in *M. edulis* plasma [236], while isoforms B, C, D and G1 were isolated from *M. galloprovincialis* hemocytes [237]. Mytilin B (PDB 2EEM) is produced from a precursor molecule, which contains an initial region (22-amino acid residues) signal peptide region, a mature peptide 34 amino acids long, followed by a C-terminal domain of 48 residues rich in acidic amino acids [236]. The assorted mytilin isoforms have been shown to possess distinctive antimicrobial activities. In fact, mytilins A, B, C, and D showed a considerable activity against both Gram-positive and Gram-negative bacteria while mytilin G1 was revealed to be functioning only against Gram-positive bacteria. Mytilins B and D have also shown potency against the filamentous fungus *Fusarium oxysporum*. Moreover, experiments for describing the kinetics of bactericidal effects showed that, at high concentrations, several hours of incubation were needed for mytilins D and G1 to kill all bacteria in contrast to the few minutes necessary in the presence of mytilin B. A potent antiviral activity was also observed for mytilin B. Therefore, the different mytilin isoforms are endowed with complementary properties, which altogether contribute to the defense mechanisms, increasing the antimicrobial potential of mussels living in the context with a high diversity of pathogens.

A cysteine-rich peptide has also been isolated from mussels (*M. galloprovincialis*) and named myticin [226]. The mature molecule is 40 residues long and shows four intra-molecular disulfide bridges. Three different isoforms of myticin have been described (A, B and C) with the isoform C being the most abundantly expressed transcript in adult mollusks. All isoforms are highly active against Gram-positive bacteria and sometimes against Gram-negative bacteria, but myticin C is also a potent antiviral compound [238,239]. Constitutively-expressed myticin C-peptides in naive mussels render oysters resistant to ostreid herpesvirus 1 (OsHV-1) infections when oyster hemocytes are incubated with mussel hemolymph. Moreover, myticin C molecules retain antiviral activity in vitro against human herpes simplex viruses 1 (HSV-1) and 2 (HSV-2), showing a high potential for biotechnological applications [240].

A strictly antifungal peptide named mytimycin (MytM) containing 12 cysteines with a molecular weight of 6.2 KDa was derived [225] from the plasma of *M. edulis*. A novel cysteine-rich peptide with noteworthy antibacterial activity was recently isolated from *Mytilus coruscus* and was named myticusin-1 [241]. This is a 104-amino acid long polypeptide including 10 cysteine residues. Antimicrobial studies showed that myticusin-1 presented a more pronounced anti-microbial activity against Gram-positive bacteria compared to Gram-negative bacteria and fungus. From the same mussel (*M. coruscus*), a novel antimicrobial peptide with 55 amino acid residues was also identified [242]. This new antimicrobial peptide is endowed by predominant activity against fungi and Gram-positive bacteria and is characterized by possessing a chitin-biding domain and by six Cys residues forming three intra-molecular disulfide bridges. The recent advent of genome sequencing technologies has also allowed the identification of two previously uncharacterized mussel AMP families, big defensins and macins. A recent analysis [243] brought to light the existence of eight novel big defensins (MgBDs) and five novel macins (mytimacins) in the transcriptome of the Mediterranean mussel *M. galloprovincialis*, therefore further extending the vast antimicrobial peptides range present in this marine bivalve organism.

The Manila clam, *V. philippinarum*, is a meaningful marine bivalve for commercial purposes, and an amino acid sequence has been identified (named VpBD) that shares common features with other AMPs, such as an α-helical structure, a net positive charge and a high hydrophobic residue ratio. The display and spacing of cysteine residues and their flanking amino acid residues indicated that VpBD represents a member of the big defensin family. The structure of big defensins, generally, comprises a highly hydrophobic region located at the N-terminal, one C-terminal cysteine-rich and positively-charged region, as well as six cysteine residues arranged to form 1–5, 2–4, 3–6 disulfide bonds in the mature peptide, in a similar pattern to mammalian β-defensins. The microbicidal activities of VpBD (expressed in *Escherichia coli*) in vitro have been investigated and demonstrated a strong antibacterial activity towards various bacterial species, namely Gram-negative (*Pseudomonas putida*) and Gram-positive (*Staphylococcus aureus*) [244]. A further big defensin has been isolated from the ark shell, *Scapharca broughtonii* [245] and clam myticin isoforms 1, 2 and 3, and clam mytilin, (similar to myticins and mytilins from mussels) have been identified and characterized in *Ruditapes decussatus* [246].

Extracts from acidified gills of the American oyster *C. virginica* [247] delivered the first defensin molecules to be purified by oysters. The peptide (named *Cv-Def*) was 38 amino acids long with six cysteines, and the molecular mass was 4.2 KDa. The antimicrobial spectrum covered by *Cv-Def* included both Gram-positive bacteria and Gram-negative bacteria [247]. Successively, three more defensins were found and characterized from the mantle, denoted as *Cg-Def*, or hemocytes, designated as *Cg-defh*1 (PDB 2B68) and *Cg-defh*2, of the Pacific oyster *C. gigas* [248,249], which shared the cystine-stabilized alpha-beta motif (CS-αβ) [250]. Other AMPs are produced in *C. gigas*, such as Cg-Prp, which belong to the family of proline-rich peptides and has been identified from hemocytes [251,252]. A 5.5-kDa antimicrobial peptide 55 amino acids long, and named *cgMolluscidin*, has been recently purified from the acidified gill extract of *C. gigas*. This sequence has no homology with any known AMPs and showed a strong antimicrobial effect against both Gram-positive bacteria (*Bacillus subtilis*, *Micrococcus luteus* and *S. aureus*) and Gram-negative bacteria (*E. coli*, *Salmonella enterica* and *Vibrio parahaemolyticus*). Finally, the last group of AMPs, described in the present review, has been identified in scallops, mainly from *A. irradians* (AiBD) and *A. purpuratus* (Ap). The scallop AiBD consisted of 531 nucleotides and produced a peptide of 122 amino acids. Recombinant AiBD was able to block the growth of both Gram-positive and Gram-negative bacteria and also presented a strong fungicidal power [253]. AP was isolated from *A. purpuratus* hemocytes, consists of 47 residues and shares partial homology with reported effective AMPs. A modified version of 30 residues designed to increase hydrophobicity and cationicity was used in antimicrobial experiments and showed an excellent activity against *Saprolegnia* sp., a parasitic pathogen fungus that attacks the culture of fish in different stages of their life, from the egg stage to grown-up animal [254].

In order to summarize all those peptides in an intelligible overview, we generated a table with their detailed characteristics (Table 4).

## 6. Applications of AMPs in Medicine and in Preventing Diseases of Aquatic Animals

In the present review, we first outlined the infectious diseases of marine bivalve and their defense mechanisms including the classification and mode of action of AMPs. In the remaining and concluding part of the review, we will focus on the potential utilization of AMPs as substitutes for antibiotics in aquaculture and in the medical field.

The economic development of several countries relies on the aquaculture of mollusks since a considerable amount of shellfish (mainly mollusks) for human consumption is produced by aquaculture. Clam, scallop, oyster and mussel farming represents a noticeable share of the aquaculture market worldwide, accounting for more than 50% of the shellfish present on the global market. The increasing demand for seafood, including bivalves, will surely extend in the forthcoming years, and higher capability productions will need to be accomplished to meet this demand. The major obstacle to the thriving of the aquaculture industry is posed by the emergence and spread of many infectious diseases, which are exacerbated by the densely-populated culture conditions in limited space. Future research needs to include the development of novel methods to control diseases in hatcheries in order to minimize the occurrences of mass mortalities caused by either obligate or opportunistic pathogens. Research efforts are mandatory to explore inexpensive and effective treatments when diseases occur in a hatchery situation to avoid production losses. Moreover, safer compounds are highly desired considering the environmental risks and that bivalve production is mainly devoted to human consumption. The possibilities to contain the spread of diseases in mollusks is not facilitated by the fact that there is limited information to fully understand the physiology of marine bivalves, in particular concerning their immune defense system. A detailed description of the immune defense mechanisms of mollusks has been provided in the first part of the review with the intent to foster the characterization of immune effectors to provide new understanding into healthiness and disease management in mollusk aquaculture. Since the introduction of antibiotics for the treatment of infectious diseases, they have been widely used in medicine and animal breeding, as well as in aquaculture with successful regimens. However, due to the overuse and misuse of antibiotics, the resistance of bacteria to antibiotics has dramatically increased, posing new challenges to human health and to the sustainability of the aquaculture industry. Moreover, the worldwide threat of a rapid increase in pathogenic multidrug-resistant (MDR) bacteria is paralleled by the environmental risks with antibiotic compounds being distributed throughout the environment. The quest for novel compounds with antibacterial activity that could overcome resistance emergence events is, therefore, an urgent societal challenge. Nature-derived AMPs are regarded as convenient templates for the development of substitutes to traditional antibiotic [255], and bivalve AMPs have been shown to be structurally different from their analogue peptides derived from the terrestrial habitat and usually present novel and unexploited structures [223,256]. AMPs’ antimicrobial activity relies on the early electrostatic interactions with the negatively-charged surface of the bacteria, therefore, free ions produced by the high salt concentrations in the surrounding medium, typical of some illnesses, could efficiently decrease interaction and antimicrobial activity. In general, marine AMPs have evolved to easily adapt to the high salt concentration of seawater, and probably, this has been obtained by the substitution of lysines with arginines. Therefore, marine bivalves are a hopeful reservoir of novel bioactive molecules for the development of alternative antimicrobials. In fact, AMPs can be regarded as encouraging candidates for selecting new and more environmentally-friendly antimicrobials. Notwithstanding the abundance of scientific knowledge on their activities in vitro, major challenges need to be outflanked to allow for their clinical application. The major obstacles to be considered are: (1) rapid degradation by proteases; (2) uncertainty of the antimicrobial activities under physiological salt, pH and serum conditions; (3) poor oral availability; (4) laborious routes of administration; (5) burdensome transportation across cell membranes; (6) non-selective receptor binding; and (7) costs associated with their production [223]. Excessive amounts of common antibiotics are used in aquaculture in some countries for both therapeutic and prophylactic purposes [257,258,259]. Moreover, veterinary antimicrobials also include compounds used clinically in human medicine [260]. Current knowledge regarding the genetic aspects of antimicrobial resistance in aquatic bacteria is highly suggestive of the possibility that antibiotics used in fish farming are likely to select antimicrobial-resistant bacteria in aquacultural environments and of their subsequent diffusion to terrestrial counterpart [261,262,263]. Therefore, in order to safeguard public health, it is of paramount importance to adopt novel methodologies used in aquaculture. The use of AMPs is one of the main strategies currently under deep investigation. It is, though, imperative to devote future efforts to design improved antimicrobial molecules with a broad spectrum of activity against a wide array of pathogenic microorganisms through the modification of native AMPs in order to achieve more selective and efficient drugs that could substitute conventional antibiotics both in aquaculture and in clinical human medicine.

One of the most awaited goals is the use of rational design to produce AMPs with improved characteristics, such as: (1) stronger antibacterial activity, (2) lower cytotoxicity and (3) ease of production on an industrial scale for obtaining a marketable drug. Native AMPs can be used as templates for the design of new antibacterial agents through peptidomimetics, where only structural key elements of the native peptide are conserved to provide a scaffold able to preserve the AMP characteristic of easy interaction with the biological target and produce an enhanced antimicrobial biological response [264]. Domains responsible for activity are, therefore, analyzed in detail and modified to obtain functionally-improved AMPs. Several authors have described the modification of primary AMP sequences to enhance their effectiveness and stability in order to obtain promising lead compounds for the development of therapeutic agents [265]. Attempt to increase AMP activities have generally involved the methodical change of amino acid residues or alternative chemical alterations, which permit the achievement of improved activities, such as: chemical modification of terminal ends of peptides [266], development of analogues containing unnatural amino acids [267], β-peptides, shortening of the native sequence, modifications of their amphipathic character, cyclization [234], hybrid peptide-peptidomimetic structures, lipidation, etc. [268]. One of the reasons for the high interest in AMPs derived from marine peptides is their ability to sustain physiological salt concentration and protease activity. In fact, a foremost role for the improvement of AMPs therapeutic impact is represented by the suitable harmonization of their hydrophobicity, amphipathicity and positive charge [269,270]. Peptides with sufficient positive charge can be modified to decrease interactions with mammalian cells, while favoring the preferential binding to bacterial cell membranes by reducing the overall hydrophobicity of the molecule [271,272]. Exploiting the variety of post-translational modifications displayed by marine AMPs could be instrumental in the design of AMPs with enhanced stability and efficacy, for therapeutic utilization in human medicine [273]. For example, the high salinity (up to 600 mM) of the marine habitat may have forged marine AMPs to be naturally endowed with a sharpened salt resistance, allowing them to conserve strong antibacterial effectiveness in relatively high-salt environments, such as in saliva, gastrointestinal fluid, serum or other body fluids [231,232]. Understanding the chemical propriety backing this salt independent activity could subside in the construction of novel AMPs that could be better endowed for facing pathogens notwithstanding the wide range of salt concentrations that could be encountered. For example, human beta defensin-1 (hBD-1) being unable to inhibit *P. aeruginosa* due to a 120 mM concentration of NaCl in the lungs of cystic fibrosis patients [274] has been engineered by constructing a chimera with β-defensin-3 (hBD-3), which shows antibacterial activity also at high salt concentrations [231,232]. The C-terminal domain of hBD-3 presents an abundance of arginine residues considered to be involved in the activity at high ionic conditions. Therefore, marine AMPs, dictating many of the universal rules featuring the ability of an AMP to inhibit microorganisms under physiological salt concentrations (120–150 mM), are of paramount importance for indicating novel strategies for the improvement of native AMP sequences [275].

## 7. Conclusions

Bivalve farming has recently reached a large portion of the fish market worldwide; therefore, from both a human health perspective and for reducing economic losses, huge efforts are devoted to the improvement of the microbiological quality of the product. We are awaiting a comprehensive understanding and knowledge of all of the infectious diseases that could affect bivalve mollusks, and insights on their defense mechanisms are only recently being deeply investigated. Several studies on bivalve immunity have expanded our understanding of the morpho-functional properties of circulating hemocytes and the humoral defense factors, but most of the patterns for microbe recognition and downhill immune pathways activation are still to be explored. At the same time, in the last decade, a large amount of data on mollusk-derived AMPs has been gathered, to allow the beginning of the initial studies to attempt to modify these lead compounds in search of a wider applicability of their antimicrobial properties in both aquaculture and human medicine.

## Figures and Tables

**Table 1:** Main viral infectious diseases of marine bivalve mollusks.

Disease (Pathogenic Agent)	Host Species	Effects on Host	Geographical Distribution	References
**VIRUSES**				
Herpes virus infection (oyster herpes virus)	Mainly hatchery-reared larvae of *Crassostrea gigas* and *Ostrea* spp.	Velar and mantle lesions; deterioration; swim in circles	Europe (France, Ireland, Italy, The Netherlands, Spain); U.K.; Australia; New Zealand, Mexico, USA, Japan, South Korea, China	[10,13,14]
Gill necrosis virus (GNV)	*Crassostrea angulata* and *C. gigas*	Destruction of gill filaments	France, Portugal, Spain, U.K.	[77]
Hemocyte infection virus (HIV)		Virus infected hemocytes	France, Spain	
Oyster velar virus disease (OVV)	*C. gigas* larvae	Larval movement affected through loss of infected epithelial cells from velum	Washington State, USA	

**Table 2:** Main bacterial infectious diseases of marine bivalve mollusks.

Disease (Pathogenic Agent)	Host Species	Effects on Host	Geographical Distribution	References
**BACTERIA**				
Larval and juvenile vibriosis (*Vibrio anguillarum*, *V. tubiashi*, *V. alginolyticus*, *V. splendidus*, *V. aestuarianus*, *V. neptunius*)	Wide range of hatchery-reared species	Tissue necrosis (due to production of exotoxin by the bacteria), up to 100% larval mortality	In all marine waters where bivalve hatchery culture is practiced	[78,79]
Brown ring disease (*Vibrio tapetis*)	*Ruditapes philippinarum*	Brown deposit on shell; degeneration of digestive gland followed by metabolic disorder and death	Entire European Atlantic coast to North Africa, including coasts of France, Portugal, Spain, Italy, U.K., Ireland and Norway, west coast of Korea	[30,32]
Roseovarius oyster disease (*Roseovarius crassostreae*)	*Crassostrea virginica* juveniles <25 mm shell length	Reduced growth rates, fragile shell development, cupping on the left valve, mantle lesions, up to 90% mortalities	USA	[37]
Pacific oyster nocardiosis (*Nocardia crassostreae*)	*Crassostrea gigas, Ostrea edulis* cultivated near infected *C. gigas*	Yellow-green pustules in the mantle, gills, adductor and cardiac muscle, up to 35% mortalities	West coast of North America from the Strait of Georgia, British Columbia to California, and Japan (Matsushima Bay), Mediterranean Sea	[39,40]

**Table 3:** Main protozoan infectious diseases of marine bivalve mollusks.

Disease (Pathogenic Agent)	Host Species	Effects on Host	Geographical Distribution	References
**PROTISTS**				
Bonamiasis (*Bonamia ostreae*, *B. exitiosa*, *B. perspora*, *B. roughleyi*)	Wide range of oyster species	Yellow discoloration of tissue, extensive lesions on gill and mantle, breakdown of connective tissue, significant mortality (up to 90%)	Europe, U.K., west coast Canada, east and west coasts of USA, New Zealand and SE Australia	[44,45,46,48,50,51,80]
Digestive gland (or Aber) disease (*Marteilia refringens*)	*Ostrea edulis* and *Mytilus galloprovincialis*	Pale digestive gland, severe emaciation, tissue necrosis, cessation of growth, mortalities up to 90% in summer	In *O. edulis*: Europe (Albania, Croatia, France, Greece, Italy, Morocco, Portugal, Spain, Sweden, Tunisia, U.K.); in *M. galloprovincialis*: northern Greece, in Italy, along the Adriatic Sea and the Campanian coast (Tyrrhenian Sea)	[56,57,58,59]
QX disease (*Marteilia sydneyi*)	*Saccostrea glomerata* and *Saccostrea* spp.	Necrosis of digestive gland, loss of condition, gonad absorption, mortalities up to 90% in summer	New South Wales, Queensland and Western Australia.	[60,61]
Dermo disease (*Perkinsus marinus*)	*Crassostrea virginica*	Severe emaciation, loss of condition, high mortality rate depending on temperature and salinity	Gulf of Mexico, southeast coast of USA, Pacific coast of Mexico, Gulf of California, Brazil	[65,66,67,68,69]

**Table 4:** AMPs isolated from marine bivalves and main characteristics.

Name	Source	Sequence	Length	Net Charge	% Hydrophobic Residues	Structure	Antimicrobial Activity	Reference
Defensin MGD-1	*Mytilus galloprovincialis*	GFGCPNNYQCHRHCKSIPGRCGGYCGGWHRLPCTCYRCG	39	5	30	Combined helix and β-sheet	Gram+	[224]
Defensin MGD-2	*Mytilus galloprovincialis*	GFGCPNNYACHQHCKSIRGYCGGYCAGWFRLRCTCYRCG	39	5	38	* Combined helix and β-sheet	Gram+ and Gram−	[227]
Mytilin A	*Mytilus edulis*	GCASRCKAKCAGRRCKGWASASFRGRCYCKCFRC	34	10	47	* Combined helix and β-sheet	Gram+ and Gram−	[225]
Mytilin B	*Mytilus edulis*	SCASRCKGHCRARRCGYYVSVLYRGRCYCKCLRC	34	9	41	Combined helix and β-sheet	Gram+ and Gram−, antiviral	[225]
Myticin A	*Mytilus galloprovincialis*	HSHACTSYWCGKFCGTASCTHYLCRVLHPGKMCACVHCSR	40	4	45	* Combined helix and β-sheet	Gram+ and Gram−; antifungal	[226]
Myticin B	*Mytilus galloprovincialis*	HPHVCTSYYCSKFCGTAGCTRYGCRNLHRGKLCFCLHCSR	40	6	37	* Combined helix and β-sheet	Gram+ and Gram−; antifungal	[226]
Myticin C	*Mytilus galloprovincialis*	QSVACTSYYCSKFCGSAGCSLYGCYLLHPGKICYCLHCSR	40	3	35	* Combined helix and β-sheet	Gram+ and Gram−; antifungal	[239]
Mytimycin	*Mytilus galloprovincialis*	MSLVLRMTLLFVVCCVVIGMSNAACCHKPFWKHCWDCTAGTPYCGYRSCNIFGCGCTCRTEPYGKSCYERGNRCRCYTDKRKRRSLSFEDISPNIKFAGLDINSDGLIEQFEFIKALEQMDIIDNTTMFHHWSIMDEDKDGTITLEEFDK	150	−2	41	* Combined helix and β-sheet	Antifungal	[225]
Mytimacin	*Mytilus galloprovincialis*	MGYIGLCGVLLSLSLLMLLQIPTSDANVLGDCWEDWSRCTRQTNWFTNIAWQSCPNRCKCQGHAGGNCIQVRSNCFLWRNKRWMCNCYGRRSGPKPGWCGF	101	7	43	* Combined helix and β-sheet	Gram+ and Gram−	[243]
Big-Defensin	*Mytilus galloprovincialis*	MNRKAILCVLYATLLIIPAPILGRVVAKKKEEKRYAAVYP IAAYAGMTVSLPVFLALVAAYGAWTVARYHIRSRSRSSSHNSHNCANNRGWCRPNCFRREYHDWYHSDTCGSYKCCRYR	119	14	42	* Combined helix and β-sheet	Gram+ and Gram−	[243]
Myticusin-1	*Mytilus coruscus*	TDHQMAQSACIGVSQDNAYASAIPRDCHGGKTCEGICADATATMDRYSDTGGPLSIARCVNAFHFYKRRGEENVSYKPFVVSWKYGVAGCFYTHCGPNFCCCIS	104	0	39	* Combined helix and β-sheet	Gram+ and Gram−, antifungal	[241]
VpBD	*Venerupis philippinarum*	LCLDQKPEMEPFRKDAQQALEPSRQRRWLHRRCLSGRGFCRAICSIFEEPVRGNIDCYFGYNCCRRMFSHYRTS	74	5	36	* Helix	Gram+ and Gram−	[244]
MCdef	*Ruditapes philippinarum*	GFGCPNDYSCSNHCRDSIGCRGGYCKYQLICTCYGCKKRRSIQE	44	4	29	* Combined helix and β-sheet	Gram+ and Gram−	[133]
VpDef	*Venerupis philippinarum*	GFGCPEDEYECHNHCKNSVGCRGGYCDAGTLRQRCTCYGCNQKGRSIQE	49	0	26	* Combined helix and β-sheet	Gram+ and Gram−	[162]
Sb-BDef1	*Scapharca broughtonii*	MTHKIVLCCIYLLLSTSFILSKHLPEERKQKKQVLLAAGA GVALSELLGPVLVGAGTLAGAALLNQAVSSNRWVIPCANNRGWCRTDCHFGEHIDDYHSD ICHSGYKCCRY	111	3	45	* Combined helix and β-sheet	Gram−	[245]
Ap	*Argopecten purpuratus*	TYMPVEEGEYIVNISYADQPKKNSPFTAKKQPGPKVDLSGVKAYGPG	47	1	25	* Polyproline rich β-sheet	Gram+, antifungal	[254]
AiBD	*Argopecten irradians*	MTRPSLVRCYSLFFTALIVMAIICPAWSEEIPKSRKKRAIPIAYVGMAVAPQVFRWLVRAYGAAAVTAAGVTLRRVINRSRSNDNHSCYGNRGWCRSSCRSYEREYRGGNLGVCGSYKCCVT	122	14	44	* Combined helix and β-sheet	Gram+ and Gram−, antifungal	[253]
AOD	*Crassostrea virginica*	GFGCPWNRYQCHSHCRSIGRLGGYCAGSLRLTCTCYRS	38	5	34	* Combined helix and β-sheet	Gram+ and Gram−	[247]
Cg-Prp	*Crassostrea gigas*	ILENLLARSTNEDREGSIFDTGPIRRPKPRPRPRPEG	37	2	21	* Proline-rich peptide	Synergistic antimicrobial activity with Cg-Def	[248]
cgMolluscidin	*Crassostrea gigas*	AATAKKGAKKADAPAKPKKATKPKSPKKAAKKAGAKKGVKRAGKKGAKKTTKAKK	55	23	29	* Helix	Gram+ and Gram−	[247]
Cg-Defh1	*Crassostrea gigas*	GFGCPRDQYKCNSHCQSIGCRAGYCDAVTLWLRCTCTDCNGKK	43	3	37	Combined helix and β-sheet	Gram+ and Gram−	[249]
Cg-Defh2	*Crassostrea gigas*	GFGCPGDQYECNRHCRSIGCRAGYCDAVTLWLRCTCTGCSGKK	43	3	37	* Combined helix and β-sheet	Gram+ and Gram−	[229]

* The predicted structures were designed using AIDA (http://ffas.burnham.org/AIDA).
